# Impact of team configuration and team stability on primary care quality

**DOI:** 10.1186/s13012-019-0864-8

**Published:** 2019-03-06

**Authors:** Sylvia J. Hysong, Amber B. Amspoker, Ashley M. Hughes, Lechauncy Woodard, Frederick L. Oswald, Laura A. Petersen, Houston F. Lester

**Affiliations:** 1 Section of Health Services Research, Department of Medicine, Baylor College of Medicine, Houston, TX USA; 2 Center for Innovations in Quality, Effectiveness and Safety (IQuESt), Michael E. DeBakey VA Medical Center, Houston, TX USA; 3 Department of Biomedical and Health Information Sciences, University of Illinois Chicago, Chicago, IL USA; 4 Department of Psychology, Rice University, Houston, TX USA

**Keywords:** Primary care teams, Quality of care, Clinical performance, Turnover, Staffing mix

## Abstract

**Background:**

The science of effective teams is well documented; far less is known, however, about how specific team configurations may impact primary care team effectiveness. Further, teams experiencing frequent personnel changes (perhaps as a consequence of poor implementation) may have difficulty delivering effective, continuous, well-coordinated care. This study aims to examine the extent to which primary care clinics in the Veterans Health Administration have implemented team configurations consistent with recommendations based on the Patient-Centered Medical Home model and the extent to which adherence to said recommendations, team stability, and role stability impact healthcare quality. Specifically, we expect to find better clinical outcomes in teams that adhere to recommended team configurations, teams whose membership and configuration are more stable over time, and teams whose clinical manager role is more stable over time.

**Methods/design:**

We will employ a combination of social network analysis and multilevel modeling to conduct a database review of variables extracted from the Veterans Health Administration’s Team Assignments Report (TAR) (one of the largest, most diverse existing national samples of primary care teams (*n*_teams_ > 7000)), as well as other employee and clinical data sources. To ensure the examination of appropriate clinical outcomes, we will enlist a team of subject matter experts to select a concise set of clear, prioritized primary care performance metrics. We will accomplish this using the Productivity Measurement and Enhancement System, an evidence-based methodology for developing and implementing performance measurement.

**Discussion:**

We are unaware of other studies of healthcare teams that consider team size, composition, and configuration longitudinally or with sample sizes of this magnitude. Results from this study can inform primary care team implementation policy and practice in both private- and public-sector clinics, such that teams are configured optimally, with adequate staffing, and the right mix of roles within the team.

**Trial registration:**

Not applicable—this study does not involve interventions on human participants.

**Electronic supplementary material:**

The online version of this article (10.1186/s13012-019-0864-8) contains supplementary material, which is available to authorized users.

## Background

The science of effective teams is well documented [1–3]; further, the National Academies’ Health and Medicine Division have recognized the importance of implementing interprofessional team-based models of healthcare to improve healthcare quality and patient-centeredness [4]. The *Joint Principles of the Patient-Centered Medical Home* (PCMH) [5] recommend a team-based approach to providing primary care to ensure that patients receive well-coordinated, high-quality care. Based on these endorsements, many primary care practices and practice networks have spent considerable resources (e.g., funds, infrastructure, and lost productivity) to reconfigure their practices into interprofessional teams with the promise of being able to deliver quality of care.

Primary care is the gateway for all other specialties in healthcare; thus, it is imperative that interprofessional primary care teams be configured to deliver the best quality care without wasting resources. However, real-world implementation often deviates significantly from the evidence-based practices advanced by the PCMH recommendations. For example, a recent study found the Veterans Health Administration (VHA) spent $774 million between 2010 and 2012 to transition to a team-based primary care model consistent with PCMH; although their investment was linked to some improvements in care outcomes [6], as of the time of the study’s writing, VA had not yet reported a positive return on investment. Findings such as these suggest better information is needed about how to best configure interprofessional teams in order to successfully implement team-based care: too few team members or the wrong type of expertise in the team may lead to reduced quality of care; too many members in a team results in wasted resources.

### Team composition and primary care performance

Teamwork is vital to providing high quality and effective patient care [7, 8]. For example, effective teamwork in primary care has been linked to improvements in distal patient outcomes such as ER utilization, thus illustrating the benefits of adhering to a team-based approach to care in the primary care setting [9]. Effective, high-performing teams exhibit certain key characteristics that partly account for their high performance [2]. For instance, the mix of personalities and values exhibited by the members of the team has been shown to enhance a team’s creativity as well as their subsequent performance on a task [10]. Team familiarity has also been reported to improve performance; teams composed of members with an established record of working together tend to form team norms and are more likely to exhibit cohesion, trust, and backup behavior, all of which can enhance the team performance [11]. Additionally, investigations of team size illustrate that team size as well as the mix of individual members’ can predict team performance, though the nature of the relationship depends on the specifics of the mix; for example, team member heterogeneity and performance are poorly correlated; however, the presence of a transformational and empowering leader improves the team performance [12]. In short, team configuration matters.

Despite rich data on the characteristics of effective teams, however, relatively little guidance is provided on how to configure the optimal healthcare team. Nelson and colleagues [13] illustrate that adherence to a team-based model of patient care (in accordance with PCMH elements in their clinics) yields multiple benefits. Specifically, they found that, compared to those who had implemented few PCMH elements, teams that had implemented more of the recommended PCMH elements (such as care coordination, shared decision-making, delegation, staffing, and team functioning) in their clinics experienced significantly higher patient satisfaction, higher performance on clinical quality measures, lower staff burnout, reduced hospitalization rates for ambulatory care, and decreased use of emergency department for primary care needs. This study suggests that closer adherence to PCMH principles as a whole can improve specific patient outcomes and clinical performance. One PCMH-based model, the Patient Aligned Care Team (PACT) [14], specifically recommends a primary care core team be composed of one provider, nurse care manager, licensed vocational nurse, and administrative clerk; however, more data are warranted to uncover which team configurations are more effective at delivering primary care.

### Team stability and quality of care

Due to current shortages in nursing and primary care provider staff [15–18], the continuity of a primary care team may prove challenging. This has raised concerns that team-based care might compromise clinicians’ ability to offer “relational continuity” to patients [19]. Indeed, difficulties in recruiting and retaining critical team members have been identified as a barrier to successfully providing team-based care [20]. Given both concerns about care continuity and the potential for reduced quality of care, it becomes increasingly important to be able to monitor team stability and assess its potential impact on quality of care.

Monitoring the stability of important roles like the provider and the nurse is also needed. Previous investigations of team roles in primary care have emphasized the importance of nursing work with one provider describing a nurse’s role as “the glue that held the clinic together” [21, 22]. In team-based models of care, nurses often serve as the care manager or patient navigator responsible for coordinating many of the services provided by the various members of the team; thus, nurses play a central role in the effective functioning of the primary care team.

### Objectives and hypotheses

The goal of this study is to determine which primary care configurations are associated with key clinical quality metrics indicative of high-quality primary care delivery, using one of the largest known samples of primary care team configuration and quality data. Our study has two specific aims:

1. Identify a set of clear, measurable, accountable, primary care performance measures, prioritized according to their overall contribution and value to primary care
2. Identify the relationships between team characteristics, team /role stability, and clinical performance. Specifically, we aim to examine the extent to which primary care teams mirror recommended team configurations and the extent to which adherence, team stability, and role stability impact healthcare quality. *We expect to find better clinical outcomes in*:

- Hypothesis 1: Teams that adhere more closely to the recommended team configurations relative to teams who deviate more egregiously.
- Hypothesis 2: Teams that are more stable over time relative to teams who are less stable
- Hypothesis 3: Teams whose clinical manager is more stable over time relative to teams whose clinical manager are less stable

## Methods

### Design

This mixed methods study combines a database review of multiple administrative and clinical data sources with a structured focus group methodology to examine the link between primary care team configurations and clinical performance via social network analysis.

### Setting: Veterans Affairs medical center primary care clinics

The proposed study will leverage clinical and administrative data sources from PACTs configured to deliver primary care at US Department of Veterans Affairs Medical Centers (VAMCs) and Community-Based Outpatient Clinics (CBOCs) nationwide. PACTs represent the VA’s operationalization and implementation of the PCMH model. VA is an ideal setting to conduct this research for several reasons. First, PCMH implementation occurred in the VHA in late 2010 and early 2011; clinics were required to staff their teams with a 3:1 full-time equivalents (FTE) ratio of primary care personnel to primary care provider, consisting of a care manager (usually a registered nurse (RN)), clinical associate (usually a licensed practical nurse (LPN)), and clerical associate (an administrative clerk). Despite this requirement and recommended configuration, PACT implementation at facilities varied considerably, resulting in a diversity of team configurations across facilities nationwide, and only 19% of teams conforming to the recommended configuration [7, 17]. The VHA maintains searchable records of the team(s) to which each individual member is assigned. We thus have a natural laboratory where many different types of team configurations, such as that found in the private sector, can be studied efficiently and effectively, while still yielding generalizable results.

Additionally, all VAMCs nationwide have used the same electronic health record (EHR) for two decades; clinical data from the EHR are uploaded into a national corporate data warehouse (CDW, a collection of databases capturing different types of clinical and administrative information). Though the databases in CDW are distinct, they contain several fields (e.g., patient ID, provider ID) that make them linkable and thus minimize the variance in the data due to the quality of information systems and data collection across over 7000 primary care teams in 1200 healthcare locations throughout the USA. Chief among the data sources available is the Team Assignments Report (see data sources section, below), which details exactly who is assigned to what team in what capacity, for every PACT in the VHA nationally. The average sample size for a study of team effectiveness is 114 teams [23]; the dataset herein constitutes a sample size nearly 70 times as large. Thus, the VA setting affords a unique opportunity to study many different types of team configurations with sufficient sample size for quantitative analysis, by using a single, standardized data source.

### Aim 1: Identifying and prioritizing primary care measures

#### Methodology overview

Before the hypotheses proposed above can be tested, appropriate outcome measures must be selected. Rather than rely on the research team’s specific expertise for measure selection, we will assemble a team of subject matter experts (SMEs) to help us identify a set of clear, measurable, accountable, primary care performance measures, prioritized according to their overall contribution and value to primary care. To ensure our expert panel navigates the available measures systematically, we will employ the Productivity Measurement and Enhancement System (ProMES), a comprehensive performance measure development approach firmly grounded in motivational theory and performance measurement principles [24, 25]. Measures are defined, weighted, and prioritized through a facilitated, structured focus group-based process. Notably, the resulting measures include non-linear functions tying specific performance levels to organizational value, thereby yielding a more nuanced approach to prioritizing work than simple linear weights, while still keeping measure scores directly comparable. Specific steps involved in identifying and measures via ProMES are detailed below.

#### Participants: design team subject matter experts

The design team will consist of up to ten SMEs, per ProMES recommendations (the terms *design team* and *expert panel* are used interchangeably herein); the team will be populated from multiple stakeholder groups, including primary care team members, primary care leadership, facility leaders (both from within and outside the VHA), and national-level policy makers.

#### Procedure

Traditional ProMES involves forming a design team of SMEs who (1) develop objectives, (2) generate performance indicators, and (3) assign value for each indicator (a process called contingency development). In this study, we will adapt the indicator development process (step 2) so that our expert panel selects from existing measures, rather than generate new ones. Each step is described below.

##### Step 1: Identify clinical performance objectives

A fundamental assumption of ProMES is that effective performance measurement cannot be accomplished without knowing what overarching performance objective is sought. The research team will facilitate a 1-day face-to-face exercise with the design team to identify the clinical objectives for primary care. According to ProMES, objectives are defined as the essential things a unit (i.e., a primary care team) does to add value to the organization. Examples of healthcare objectives include patient care performed according to quality standards, effective patient throughput, and personnel allocation matched to patient workload. The key question the design team will answer is “what is the primary care team trying to accomplish when it delivers care?” The facilitators will guide the design team to arrive at three to six objectives that meet the following criteria: (1) is stated in clear terms; (2) designed so that if exactly that objective was accomplished, the facility would benefit; (3) the set of objectives covers all important aspects of clinical care; (4) is consistent with broader objectives (such as Agency for Healthcare Research and Quality [AHRQ] conceptualizations of primary care); and (5) leadership is committed to each objective. Objectives will be compared against these criteria to ensure their utility in facilitating the subsequent steps of the process.

##### Step 2: Select performance indicators

Having identified primary care teams’ healthcare delivery objectives, the next step is typically to identify indicators for these objectives. For each objective, the expert panel answers the following question: “How would you show that the primary care team is meeting the stated objective?” To accomplish this, the expert panel will review a document containing names, sources, and operational definitions of all outpatient clinical performance measures currently tracked by VA. This inclusion criterion is necessary to ensure the resulting metrics are available for analyses in aim 2 of the proposed work; nonetheless, to ensure generalizability beyond the VA healthcare system, any metrics that capture VA-specific processes or policies (e.g., homeless veteran metrics; veteran patient portal usage) will be excluded. Metrics will be drawn from VHA’s principal outpatient clinical data sources: the Healthcare Effectiveness Data and Information Set (HEDIS) [26], which is used by most medical practices nationwide; the Strategic Analytics for Improvement and Learning (SAIL) metrics [27], and VA’s Electronic Quality Measures (eQM), which provides, real-time, 100% sampling of patients for metric calculation. After identifying objectives in the face-to-face meeting, the team will meet virtually in a series of facilitator-led meetings for up to approximately 12 h of meeting time; for each objective, the design team will select a set of performance indicators that capture the extent to which the primary care objectives are being achieved.

##### Step 3: Prioritize indicators by developing contingencies

Indicators provide information about *what* is valued in performance (e.g., the lag between when a clinical test is ordered and an appointment scheduled signals timeliness in care delivery); however, *what level* of performance is acceptable, or *how much* a given level of improvement is valued (Is an average of 7 days acceptable? How much worse is 8 days? 10? How much better is 5 days?), is captured by a ProMES tool called contingencies.

**The value of contingencies**

Contingency development generates a function for each indicator that shows how much the different amounts of the indicator (e.g., 5 vs. 7 days) contribute to overall effectiveness (in this case PACT effectiveness). By relating each indicator to overall effectiveness, they are put on the same measuring scale, which ranges from − 100 to + 100. Thus, the various indicators can be directly compared, prioritized, and combined into a single measure if needed. Most importantly, it reflects an explicit statement of what elements of coordination are valued, and what level of coordination-related performance is expected and valued by the PACT and the facility.

**Creating contingencies**

After the indicators are developed, the design team will convene for up to six to eight additional hours in multiple virtual meetings to develop a contingency function for each indicator. The expert panel must identify the maximum and minimum possible levels of performance, the minimum expected performance level, and scale the various levels of performance to a common metric of effectiveness for each indicator. Pritchard and colleagues explain this protocol in detail [24]; a summary can be found in Additional file 1.

#### Data Analysis

No data analysis occurs for this aim other than compiling the list of indicators and their respective contingency functions.

### Aim 2: Identify the relationships between team characteristics, team/role stability, and clinical performance

#### Overview: social network analysis as an approach to studying team configuration

Social network analysis (SNA) investigates social structures by characterizing and analyzing structures in terms of nodes (individual actors, people, or things within the network) and the ties, edges, or links (relationships or interactions) that connect them. In this study, we propose to examine three distributional properties of social networks (in this case, primary care teams) to answer the fundamental questions about staffing mix and its relationship to team-level clinical performance: average *degree* (i.e., the number of teams of which each member is a part, then averaged across all members in the team), average *betweenness* (i.e., the number of people that members of a team uniquely bridge, then averaged across all members in the team), and the *Blau Index* (i.e., the extent to which there is balance across the four team roles [primary care provider, registered nurse care manager, clerical associate, and licensed practical nurse]).

#### Data sources

Data will be sourced from multiple existing datasets available through the VA Service Support Center (VSSC), a centralized collection of databases and portals curated by VHA.

##### Primary care almanac team assignments report

Updated nightly, the Primary Care Almanac Team Assignments Report (TAR) lists all active PACTs at every healthcare location within the VHA system, and the names and roles of the staff members in each team.

##### HR smart

HR Smart is the VHA’s official Employee Data Cube, housing employee information for all VHA employees, including demographics (e.g., gender, age), occupational characteristics (e.g., highest degree, job title, current role), and tenure history within the VHA (e.g., start and termination dates with the Federal Government, within the Veterans Health Administration, and within each VHA facility).

##### Electronic quality measures

Curated by VHA’s Office of Organizational Excellence, electronic quality measure (eQM) relies on nationwide, automated extraction of data pooled in the VHA Corporate Data Warehouse (CDW) to generate near real-time, full-population measures of clinical performance. The advantage of the eQMs over other systems, such as VA’s External Peer Review Program (EPRP), is that scores can be calculated using the entire patient population (100% sampling) rather than estimating scores based on a sample of abstracted records, thereby eliminating both sampling and missing data concerns. This information is updated daily so that users can always access the most current measure information [28–30]. For this study, we will draw monthly extracts.

##### Healthcare Effectiveness Data and Information Set metrics

HEDIS is a tool used by more than 90% of America’s health plans to measure performance on important dimensions of care and service. Altogether, HEDIS consists of 81 measures across 5 domains of care, including effectiveness of care (i.e., clinical performance). HEDIS Metrics for VHA facilities are available through the SAIL reporting system (see below).

##### Strategic Analytics for Improvement and Learning

SAIL is a system for summarizing hospital system performance within Veterans Health Administration (VHA) modeled after the Truven Analytics Top Health Systems study [27]. SAIL assesses 26 quality measures in areas such as death rate, complications, and patient satisfaction, as well as overall efficiency at individual VA Medical Centers (VAMCs). SAIL data tables are updated every quarter.

#### Measures

##### Team configuration characteristics

We will employ SNA to determine the extent to which actual and ideal team membership configurations are aligned (described in the introduction and detailed below). Following a similar methodology used in prior research [9], we will examine the following team network characteristics: average *degree* (the number of teams of which each member is a part, averaged across all members of the team), average *betweenness* (the number of people for which a given member serves as the only bridge or connection, averaged across all members of the team), and the *Blau Index* (the extent to which there is a balanced role representation in the team).

##### Adherence to recommended team configuration

For a given observation period, we will calculate an index of deviation from the ideal team configuration using the aforementioned SNA configural properties of teams. Mathematically, a team with exactly one provider, care manager, clinical associate, and clerical associate, respectively (i.e., the configuration recommended by VHA as the optimal for primary care teams within the VA system), has an average degree of 1 (i.e., each member assigned to one and only one team), an average betweenness of 0 (members of the team do not connect to members of other PACTs), and a Blau Index of .75 (there are four members in the PACT, each with a unique role: provider, care manager, clinical associate, and clerical associate). For each team, we will calculate each property, subtract each from its respective recommended score, and use this as a team deviation score for each property. Each property may reveal unique information, and a positive deviation from an ideal configuration may imply something different than a negative deviation. Thus, to capture these nuances, each property will be entered separately in our analysis, rather than calculating a composite measure of adherence from the three properties.

##### Team stability

For a given observation period, we will calculate a team stability coefficient for each team, operationalized as:

$$ 1-\left(\frac{n\ \mathrm{of}\ \mathrm{separations}\ \mathrm{during}\ \mathrm{observation}\ \mathrm{period}}{\mathrm{average}\ \mathrm{n}\ \mathrm{of}\ \mathrm{team}\ \mathrm{members}\ \mathrm{during}\ \mathrm{observation}\ \mathrm{period}}\right) $$

This coefficient accounts for instability in the team due to many changes in personnel and/or large amounts of time with positions unfilled. A value of 1 denotes complete stability (no separations occurred); decreasing positive values between 1 and 0 denote increasing instability due to increasing separations relative to the average team size; increasing negative values denote increasing instability due to more personnel changes than average team members (such as when the team is understaffed).

##### Role stability

Two facets, each to be calculated separately, comprise role stability: *staffing sufficiency*, i.e., the percent of time a given role remains filled in the team, and *role continuity*, which captures changes in who fills a given role over a given observation period. For each role, we will operationalize staffing sufficiency as:

$$ \left(\frac{n\ \mathrm{of}\ \mathrm{time}\ \mathrm{points}\ \mathrm{role}\ \mathrm{was}\ \mathrm{filled}\ \mathrm{during}\ \mathrm{observation}\ \mathrm{period}}{n\ \mathrm{of}\ \mathrm{time}\ \mathrm{points}\ \mathrm{in}\ \mathrm{observation}\ \mathrm{period}}\right) $$

A value of 0 denotes the position was never filled during the observation period, whereas a value of 1 denotes the role was filled throughout the entire observation period.

For each role, we will operationalize role continuity as:

$$ \left(\frac{n\ \mathrm{of}\ \mathrm{within}-\mathrm{role}\ \mathrm{separations}\ \mathrm{during}\ \mathrm{observation}\ \mathrm{period}}{n\ \mathrm{of}\ \mathrm{time}\ \mathrm{points}\ \mathrm{in}\ \mathrm{observation}\ \mathrm{period}}\right) $$

A value of 0 denotes no continuity (i.e., as many individuals in a role as available time points in the observation), whereas a value of 1 denotes complete continuity (only 1 individual filling the role throughout the observation period). Data for team and role stability calculations will be obtained from the TAR. Table 1 shows a worked example.

**Table 1:** Worked example of team stability, staffing sufficiency, and role continuity calculations

Role	Time point in study observation period						*n* of within role separations in obs. period	*n* of time points role filled	Staffing sufficiency	Role continuity
	1	2	3	4	5	6				
PCP	a	a	a	0	0	b	1	4.00	.67	0.83
Care manager	c	c	c	c	c	c	0	6.00	1.00	1.00
Clinical associate	d	e	e	e	f	f	2	6.00	1.00	0.67
Clerical associate	g	g	0	0	0	0	0	2.00	.33	1.00
*n* of roles	4	4	3	2	2	3				
Calculating team stability:										
A. *n* of separations during the observation period: (i.e., total number of people who left their role, whether or not they were replaced by a new team member)										4
B. Average *n* of roles during the observation period										3
Coefficient of team stability (1 − (A/B))										− .33

##### Team size covariates

To account more directly for variations team size, we will calculate from the TAR the total number of members and the total number of full-time equivalents (FTE) in a given team at each time point.

##### Clinical performance

Clinical performance will be measured as specified by the outcomes of the work described in aim 1. Clinical performance data will be collected monthly from the data sources described in the previous section.

#### Sample size

The most current data from the TAR indicates a total of 7700 primary care teams nationwide. Teams that do not directly focus on primary care (e.g., spinal cord injury teams), as well as teams created for educational purposes (e.g., resident-led teams), will be excluded. We expect this number to be relatively small, less than 10% of the total available teams. This will leave over 5000 teams, distributed across 152 VA Medical Centers and approximately 1400 community-based outpatient clinics, for use in our analyses.

#### Procedure

The first step will be to obtain the needed data from each of the sources detailed in the “Data sources” section above. The various datasets will then be imported into a SQL-friendly database platform and linked into a single data file to prepare the dataset for analysis. The TAR will be the primary link between the clinical performance data source and the turnover source. Clinical performance data is available at the patient level but aggregable to the team level via the Provider ID, which is present in the TAR and can be cross-referenced with the Team ID. Turnover data is available at the individual employee level and indexed by employee ID, which is also available in the TAR, thus making it possible to link employee data, clinical performance data, and team configuration data. All team configuration measures will be calculated as described in the “Measures” section above from fields in the TAR. This process will be carried out monthly for a period of 1 year in order to generate the time-based variables and time points required for the longitudinal hypothesis tests. The year-long study observation period will consist of 12 time points (1 time point = 1 month) beginning upon completion of aim 1, which we expect will occur at the end of the first study year.

#### Analysis

Hypothesis 1 predicts that teams adhering more closely to recommended team configurations are more likely to exhibit higher clinical performance scores than those who deviate from the recommended team configuration (the higher the deviation, the lower the predicted performance). To test this hypothesis, we will conduct a multilevel model (MLM), with team-level clinical performance as the outcome; time as a level-1 covariate; the following variables as team-level (level 2) predictors: deviation from ideal average degree; deviation from ideal average betweenness; and deviation from ideal Blau Index, and the following variables as team-level covariates: total team FTE, number of team members in the team. We will calculate intra-class correlation coefficients (ICCs) to assess the level of dependency of teams within facilities on clinical performance. If ICCs > .05, then we will account for the dependency of teams within facilities by adding facility as a level-3 nesting variable [31]. Our hypothesis will be supported by significant effects of deviation from ideal network properties (average degree, average betweenness, and Blau Index).

Hypothesis 2 predicts that teams who are more stable over time are more likely to exhibit higher clinical performance scores than those who are less stable over time. We will test a model with clinical performance as the outcome; time, change in the number of team members at each time point from the previous time point, level-1 predictors with time *x* − 1 performance as a time-varying covariate (i.e., an autoregressive covariance structure); and facility as a level-2 predictor to account for nesting. Our hypothesis will be supported by a significant effect of the number of team members at each time point and of team stability during the observation period.

Hypothesis 3 predicts that teams whose nurse care manager is more stable over time will exhibit higher clinical performance scores than those whose clinical manager is less stable over time. We will test an MLM with clinical performance as the outcome measure; time and change in RN sufficiency and RN continuity at each time point from the previous time point (role stability) as level-1 predictors, with time *x* − 1 performance as a time-varying covariate; and facility as a level-2 predictor to account for nesting. Our hypothesis will be supported by a significant effect of RN sufficiency and RN continuity during the observation period.

We will calculate intra-class correlation coefficients (ICCs) to assess the level of dependency of teams within facilities on clinical performance. If ICCs > .05, then we will account for teams within facilities by including facilities as a level-3 nesting variable in tests of all hypotheses.

## Study status

We have received IRB approval; our initial meeting with the design team was completed on November 29, 2018, where we identified objectives for primary care. Virtual meetings for selecting indicators with the design team (in support of aim 1) will begin in January 2019. We are also in the process of securing data use agreements to obtain the data for aim 2.

## Discussion

### Contributions to practice

The forecasted results of our study could inform numerous changes in team-based primary care practice. First, our study will yield a strategically targeted and prioritized set of performance measures for monitoring primary care performance from the myriad measures currently available. The value in the set resulting from our study compared to other measure set initiatives comes from the measure prioritization. The measures will be not only rank-ordered, but also weighted statistically in terms of their contribution for meeting organization and team-based objectives. Further, each measure will have assessments of value for key levels of performance. Primary care in both the public and private sectors can thus rely on a nimble, targeted set of metrics and an understanding of how the phenomena they capture relate to the overall mission of primary care.

Second, our study’s findings can inform staffing of primary care teams in both private- and public-sector clinics such that teams are configured optimally, with adequate staffing, and with the right mix of roles within the team. Our findings will test whether recommended staffing ratios and configurations currently implemented in VHA based on PCMH principles are associated with better clinical performance; this will help inform facilities in the public and private sector the extent to which such staffing models can generalize to their situation.

### Limitations

Although the study data allow us to test whether a specific configuration yields a better performance on quality scores than other configurations, as well as whether high performing teams seem to have commonalities in how they are configured, we are limited by the available data. Any configural changes (and consequently, changes in performance) occurring at a finer periodicity than the monthly snapshots we are capturing will not be detectable by our methods. Further, this study relies exclusively on existing, archival data to test its hypotheses. Thus, we have no data on the quality of the actual functioning team, given a specific configuration (for example, two teams with the ideal recommendation could function very differently, depending on the relationships and the mental models formed by the team). We can only hypothesize that the observed changes in the stability of a given team could potentially be driven by team-functioning factors. Future studies could employ more traditional social network analysis survey methods to explore this issue more fully.

### Contributions to science

This study stands to contribute to the scientific literature on team composition. Research suggests that increasing staff members is associated with improved patient care; however, we are not aware of other studies that consider appropriate team size, composition, and configuration longitudinally or with sample sizes such as those within the proposed study. The study is thus uniquely poised to garner new insight into the true nature of team composition within primary care teams.

## Additional file

Additional file 1: Technical Appendix: the Productivity Measurement and Enhancement System. (DOCX 47 kb)
