# Supplementary material for: Impact of team configuration and team stability on primary care quality
Source: Implement Sci. 2019 Mar 6;14:22. doi: 10.1186/s13012-019-0864-8 (PMC6404317; doi:10.1186/s13012-019-0864-8)
Supplement: Supplementary file 1 — Technical Appendix: the Productivity Measurement and Enhancement System. (DOCX 47 kb) [file 13012_2019_864_MOESM1_ESM.docx]

# Technical Appendix: The Productivity Measurement and Enhancement System

This appendix describes the basic steps for conducting ProMES in a unit or department, and provides evaluation criteria for assessing the quality of objectives and indicators. An exhaustive treatise of design considerations and implementation issues is beyond the scope of this appendix. The reader is referred to Pritchard, Weaver & Ashwood (2011)^[[1]](#footnote-1)^ for such a purpose.

## **ProMES’ Steps to Motivationally-Driven Performance** Measurement

### Step 1: Select the Design Team

The first step in ProMES is to form the team that will participate in the ProMES process (the design team), to develop measures of performance. Ideally, design teams consist of 6-8 representatives from an intact work group or department (preferably of varying roles), the supervisor of the workgroup, and 1-2 facilitators. Facilitators serve as independent helpers of the process, rather than representatives of any given party or discipline on the design team, and are usually not member of the work group.

### Step 2: Identify Objectives

Consistent with goal setting theory and empirical research in that domain, a fundamental element of ProMES is that performance becomes more measurable and improves when performers clearly understand the overarching performance objectives toward which they are striving. Creating a cohesive performance measurement system thus requires identifying clear performance objectives. According to ProMES, objectives are defined as the essential things a unit (e.g., a health care facility, a medical service) does to add value to the organization; in other words, objectives are the main result (and associated characteristics) of the work of the unit. Examples of health care objectives include: patient care performed according to quality standards, effective patient throughput, personnel allocation matched to patient work load. Health care objectives answer the question, “what are we trying to accomplish when delivering care?” For objectives to be useful, they must meet the following criteria: (1) stated in clear terms, (2) designed so that if exactly that objective was accomplished, the facility would benefit; (3) the set of objectives cover all important aspects of performance; (4) consistent with broader facility objectives; (5) leadership is committed to each objective.

### Step 3: Develop Performance Indicators

Having identified the unit’s objectives, the next step is to develop performance indicators for these objectives. For each objective the design team answers the following question: “How would you show that the stated objective is being met?” To accomplish this, the design team usually holds a series of meetings to identify, for each objective, a set of performance indicators that capture the extent to which the coordination objectives are being achieved. Each indicator must meet multiple criteria regarding its validity, comprehensiveness, impact, feasibility, and usability (see Appendix A). Once the indicators have been completed to the satisfaction of the group, the next step is to obtain formal approval of the objectives and indicators from leadership. This essential step has multiple purposes including checking accuracy and completeness, but most importantly, ensuring alignment with leadership objectives and securing buy-in.

### Step 4: Prioritize Indicators by Developing Contingencies

Indicators provide information about what is valued in coordination (e.g., the number of days between an abnormal fecal occult blood test (FOBT) result and a scheduled colonoscopy appointment signals timeliness in care delivery); however, what level of performance is acceptable, and how much a given level of improvement is valued (e.g., is an average of 7 days acceptable? How much worse is 8 days? 10? How much better is 5 days?) is provided by a ProMES tool called contingencies. Contingency development generates a function for each indicator that shows how much the different amounts of the indicator (e.g., 5 vs. 7 days) contribute to overall effectiveness (in this case primary care coordination effectiveness). For each indicator the design team identifies the following values:

- *Maximum performance level*: maximum feasible value under ideal situations
- *Minimum performance level:* the score where upper management will feel concerned
- *Minimum expected Level (Zero Point):* neither good or bad; just meets minimum performance expectations

These levels are then scaled to a common metric of effectiveness:

- *Maximum effectiveness score:* Value to the organization (0-100) associated with the maximum performance level (whichever indicator is most important always has a value of 100 assigned to it)
- *Minimum effectiveness score:* Value to the organization (0-100) associated with the minimum performance level

Figure A below presents a worked example of a sample performance indicator for primary care team coordination, the percentage of appointments in a given month for a given team that started on time.^^[[2]](#footnote-2)^^

By relating each indicator to overall effectiveness, they are put on the same measuring scale, which ranges from -100 to +100. Thus, the various indicators can be directly compared, prioritized, and combined into a single measure if needed.

Most importantly, this method reflects an explicit statement of what elements of coordination are valued, and what level of coordination-related performance is expected and valued by the primary care delivery team and the facility. Pritchard and colleagues explain contingency development in detail. Once contingencies are developed, the design team obtains formal approval of the contingencies from leadership, in the same manner and for the same reasons as the performance indicators.

| 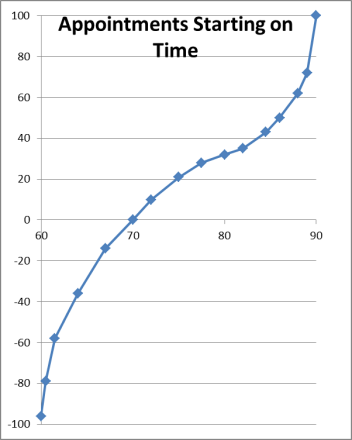 | **Key Value Description** | **Value** |
| --- | --- | --- |
|  | Maximum performance level | 90% |
|  | Minimum performance level | 60% |
|  | Minimum expected level (zero point) | 70% |
|  | Maximum effectiveness score | +100 |
|  | Minimum effectiveness score | -100 |

- Figure A. Key Values and Contingency Curve for Sample Indicator (percent of Appointments Starting on Time)

### Step 5: Provide Feedback Reports

According to Pritchard and colleagues the feedback system (steps 5 and 6, providing feedback reports and holding feedback meetings) is considered the most important part of ProMES. As such, the design of the feedback report becomes critical and should follow evidence-based principles of good feedback design {Hysong, 2009 #4125;Hysong, 2006 #3734;Hysong, 2016 #4813}. Consistent with such principles, Pritchard and colleagues recommend feedback should be provided as frequently as the natural cycle of the work being evaluated, and as frequently as is feasible to collect the required data to generate the feedback report. Feedback should be disseminated as soon as possible after the completion of the reporting period, and include both numeric and graphical information. In the study serving as our case example, the natural periodicity of data availability was monthly, making it the natural periodicity for delivering feedback reports.

According to Pritchard and colleagues, the content of the feedback report should include scores for each indicator along with their corresponding effectiveness. In addition to the current month’s data, including historical data as well as priorities for next period. These design principles were all taken into consideration when designing the coordination dashboard for our case example study. Appendix B presents a fully annotated example of the feedback report used.

### Step 6: Feedback Meetings

Feedback meetings should soon follow dissemination of reports to discuss their content, ideally held by the supervisor, though ProMES facilitators are recommended for the first few meetings to ensure they are conducted appropriately. All members of the unit, not just the design team, should be involved. The purpose of the meeting is to review the performance scores on the report and identify both facilitators and barriers to improvement. In the case example study we specifically adopted the Team Dimensional Training (TDT) <ref> approach to debriefing, which is consistent with ProMES recommendations and provides specific structure: By the end of the meeting, teams are to have identified at least two things that they can start doing, stop doing, or continue doing to improve their performance, in this case coordination.

**Step 7: Periodically Review Systems**

The last step simply involves building in periodic check-ins to determine whether any measures need to be added, changed, or deleted, or whether the value (and thus the contingency function) of any existing measures requires modification. Pritchard and colleagues recommend a minimum of 12-14 time points before to observe a sustainable improvement; a review before then would be counterproductive. Because the research study only captured seven time points, no periodic review was carried out.

## Measurement Criteria For ProMES Objectives and Performance Indicators

### Criteria for ProMES objectives

Below are the criteria used to evaluate the quality of performance objectives according to the ProMES model. Objectives that meet these criteria are important prerequisites for identifying and developing appropriate performance measures.

- Objectives should be stated in clear terms
- Objectives should be designed so that if exactly that objective was accomplished, the organization would benefit
- The set of objectives must cover all important aspects of the work (in our case, all important aspects of coordination)
- Objectives must be consistent with the objectives of the broader organization
- Leadership must be committed to each objective
- The number of objectives should be manageable, normally 3 to 8.

### Criteria for ProMES Indicators

Below are the criteria used to evaluate the quality of a performance measure according to the ProMES model. For consistency with more health-care specific model, they are organized according to the criteria used by the National Quality Forum to evaluate clinical measures.

#### Validity/Reliability

- Indicators must validly measure the objective
- Indicators must be largely under the control of unit personnel
- The information provided by the indicator must be neither too general nor too specific.

#### Comprehensiveness (not part of NQF criteria)

- All important aspects of each objective must be covered by the *set* of indicators

#### Impact (Value)

- Indicators must be consistent with the objectives of the broader organization
- Indicators should be designed so that if the indicator was maximized (i.e., perfect score), the organization would benefit (value – similar to NQF’s Impact)

#### Feasibility

- Leadership must be committed to *each* indicator
- Accurate indicator data must be cost effective to collect

#### Usability

- Indicators must be understandable and meaningful to unit personnel
- It must be possible to provide information on the indicator in a timely manner

1. Pritchard RD, Weaver SJ, Ashwood EL. Evidence-based productivity improvement: A practical guide to the productivity measurement and enhancement system. New York: Routledge Academic.; 2011. [↑](#footnote-ref-1)
2. Source: Hysong, S.J. and Petersen, L.A. (2013-2019). *Identifying and Delivering Point-of-care Information to Improve Care Coordination.* VA Health Services Research and Development grant no. CRE 12-035. [↑](#footnote-ref-2)
